# Assessment of App Quality, User Experience, and Willingness to Recommend a Digital Therapeutic for Patients With Axial Spondyloarthritis: Subanalysis of a 12-Week Pretest-Posttest Intervention Study

**DOI:** 10.2196/88949

**Published:** 2026-10-26

**Authors:** Hannah Labinsky, Amelie Wuest, Patricia Possler, Tobias Heusinger, Maxime le Maire, Anna Fleischer, Karsten Sebastian Luetkens, Michael Gernert, Ottar Gadeholt, Robert Leppich, Astrid Schmieder, Ludwig Hammel, Billy Sperlich, Matthias Fröhlich, Marc Schmalzing, Patrick-Pascal Strunz

**Affiliations:** 1 University Hospital Würzburg, Department of Internal Medicine 2, Rheumatology/Clinical Immunology Wuerzburg, Bavaria Germany; 2 University of Würzburg, Medical Faculty Wuerzburg Germany; 3 University Hospital Würzburg, Department of Internal Medicine 2, Psychosomatic Medicine Wuerzburg Germany; 4 University Hospital Würzburg, Department of Diagnostic and Interventional Radiology Wuerzburg Germany; 5 Chair of Software Engineering (Informatik II), Department of Computer Science, University of Würzburg Wuerzburg Germany; 6 University Hospital Würzburg, Department of Dermatology, Venereology, and Allergology Wuerzburg Germany; 7 Deutsche Vereinigung Morbus Bechterew e. V. Schweinfurt Germany; 8 Integrative and Experimental Exercise Science and Training, Institute for Sports Science, University of Wuerzburg Wuerzburg Germany

**Keywords:** mobile health, mHealth, digital therapeutics, gamification, reinforcement, mobile apps

## Abstract

**Background:**

Axial spondyloarthritis (axSpA) is a chronic inflammatory rheumatic disease affecting the axial skeleton that requires regular physical exercise in addition to pharmacotherapy. However, a significant proportion of patients do not exercise frequently enough. Axia is a digital therapeutic (DTx) that offers a solution by providing a disease-specific and patient-tailored home-based exercise program for patients with axSpA. The app combines structured exercise with patient education and disease management features and incorporates gamification and behavioral conditioning to promote user engagement. However, it has remained unclear how Axia is perceived and evaluated by patients.

**Objective:**

This study aimed to describe app quality, user experience, and willingness to recommend the app to others after 12 weeks of use by applying standardized questionnaires.

**Methods:**

This single-center study was conducted at an outpatient tertiary clinic. After 12 weeks of ad libitum app use, 27 participants with axSpA completed paper-based assessments of the user version of the Mobile App Rating Scale (uMARS; range 1-5) and the net promoter score (NPS; range −100% to +100%).

**Results:**

The mean overall uMARS app quality score was 4.44 (SD 0.43). Functionality received the highest mean score (4.54, SD 0.53), whereas information received the lowest (4.34, SD 0.49). The mean NPS was 9.52 (SD 0.75), corresponding to an overall NPS of 93%. The uMARS overall quality score correlated strongly with the NPS (ρ=0.57; *P*=.002). Except for physical therapy at baseline, no other baseline characteristic or therapeutic effect correlated with either the uMARS or NPS.

**Conclusions:**

Axia was perceived as a user-friendly DTx after 12 weeks of use. The findings support the acceptability of disease-specific DTx for patients with axSpA and the potential integration of DTx into routine care.

## Introduction

### Background

Axial spondyloarthritis (axSpA) is an inflammatory rheumatic disease characterized by chronic inflammation of the axial skeleton [1]. In addition to anti-inflammatory pharmacotherapy, regular disease-specific exercise and patient education represent the second major pillar of axSpA management and are strongly recommended in international guidelines [2]. Disease-specific home-based exercise programs are one recommended approach to therapeutic exercise and have been shown to improve disease activity, physical function, and quality of life in axSpA [3]. Despite these strong recommendations, many patients with axSpA exercise insufficiently [4]. In the past, home-based exercise has been mainly provided through paper-based brochures and booklets or through video CDs or DVDs [3,5,6].

The widespread availability of smartphones and the increasing use of mobile exercise apps for home-based exercise provide an opportunity to offer axSpA-specific digital therapeutic (DTx) to support home-based exercise [7]. Regular use of such a DTx could help close this existing gap in patient care by motivating and guiding patients to exercise more frequently [7]. However, rapid attrition and declining user engagement remain challenging issues for many DTx [8,9].

To bridge this current gap in care, we developed a medical app named Axia focusing on home-based exercise and disease management [7]. A strong focus on gamification and motivational elements might help to mitigate attrition [8]. Axia therefore includes gamification elements that may support permanent user engagement during home-based exercise. In a recently published randomized controlled trial (RCT), the Bechterew-App Trial I with the medical app Axia, patient-perceived disease activity, functional status, and quality of life significantly improved [10]. Axia has recently been approved as an official digital health application (DHA; in German DiGA for *Digitale Gesundheitsanwendung*) for axSpA in Germany based on the data from this RCT [11].

However, due to the fully remote nature of this RCT, no objective outcomes have been assessed so far. Furthermore, app quality, user experience, and willingness to recommend the app have not previously been assessed using standardized and validated questionnaires. High user satisfaction and recommendation rates, as indicators of acceptance, are crucial quality indicators for DTx, as they may support high and sustained user engagement [8,12]. Acceptance may facilitate continuous engagement and thereby treatment adherence, both of which are essential for a DTx to exert its therapeutic effect [12]. The user version of the Mobile App Rating Scale (uMARS) and the net promoter score (NPS) are established and validated instruments for assessing app quality, user experience, and likelihood of recommending the intervention to others [13,14]. Both have been widely used to evaluate DTx for inflammatory rheumatic diseases [15-18].

### Objectives

A second interventional trial, the Bechterew-App Trial II, was initiated to further evaluate the effects of Axia on biomechanical outcomes and additional patient-reported outcomes that were not assessed in the original RCT [10]. As a substudy of this parent trial, we aimed to assess the uMARS and NPS for Axia to assess app quality, user experience, and willingness to recommend the app and make these results comparable with those of other DTx.

## Methods

### Study Design

This study was conducted as a substudy of the Bechterew-App Trial II. Figure 1 provides an overview of the study design. Data from the parent trial have already been published elsewhere [19]. This study aimed to evaluate app quality, user experience, and recommendation rates for Axia after 12 weeks of app access using standardized and validated questionnaires. It was not designed to isolate the specific contribution of individual app components, including gamification. Following a 12-week observational period under standard-of-care conditions, participants received access to Axia and were able to use the app according to their personal preferences for an additional 12-week period. After this use period, participants completed a survey that included the German versions of the uMARS and NPS. The study commenced in February 2024, and the last participant completed the survey on July 7, 2025. This study was registered in the German Clinical Trials Register (Deutsches Register für Klinische Studien; DRKS00038067). A STROBE (Strengthening the Reporting of Observational Studies in Epidemiology) statement checklist is provided in Multimedia Appendix 1 [20].

**Figure 1:**
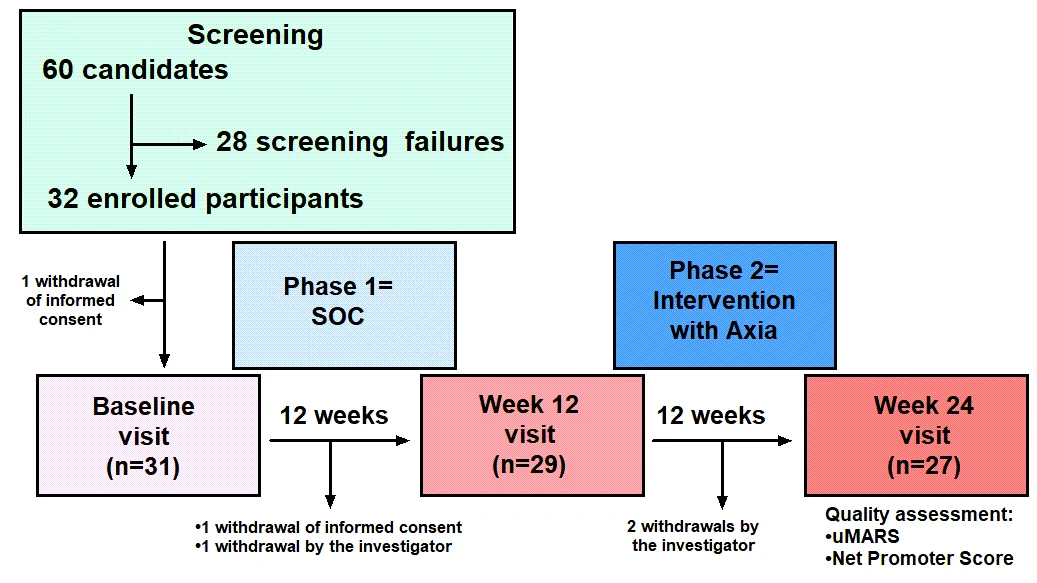
Study design and study flow. SOC: standard of care; uMARS: user version of the Mobile App Rating Scale.

### Intervention and Setting

Axia is a class I medical device that has been developed by Applimeda GmbH. Clinical and patient-representative input was provided by the University Hospital of Würzburg and the German patient self-help organization Deutsche Vereinigung Morbus Bechterew. During the study period, the app was available as a German-language app only and could be used on the common smartphone operating systems. Axia has been approved as a DHA for axSpA since the end of January 2026. The app provides home-based exercise programs tailored to patients with axSpA. In addition to structured exercise sessions, users can record short daily exercise routines, sports activities, and step goals. The intervention also includes educational content related to disease management and physical activity. Axia contains several engagement-supporting features, including an activity-based point system, milestones, streaks, achievement tracking, push notifications, and an animated mascot. These gamification features are shown in Figure 2.

**Figure 2:**
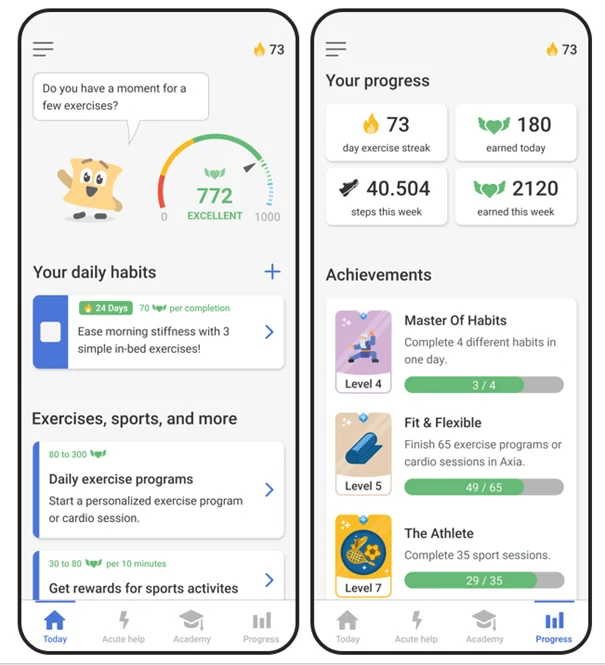
Central elements of gamification implemented on the Axia app: home screen and graphical user interface of Axia. One gamification element is an avatar that interacts with the user by adapting its mood to the user’s physical activity level and app use (top left). The avatar also appears in push notifications sent to the user. Another gamification element is a point gauge resembling a speedometer. On the right panel, users can earn streaks and achievements by exercising regularly with the app or by engaging with the educational features. Points are also awarded for use of the educational content.

### Participants and Eligibility Criteria

Participants were adults (≥18 years) with a confirmed diagnosis of axSpA according to the Assessment of SpondyloArthritis International Society (ASAS) classification criteria and theoretically able to use Axia for daily exercise [21]. Disease activity, assessed via a Bath Ankylosing Spondylitis Disease Activity Index (BASDAI) of at least 3.0, was necessary for inclusion in the trial, as well as German-language proficiency and adequate digital literacy, which was defined as the regular use of a commonly used messenger service. Major exclusion criteria were medical contraindications to exercise, pregnancy, use of other therapeutic apps for axSpA, and any condition preventing safe participation in the exercise program. The supplementary file of the study by Strunz et al [19] provides a comprehensive overview of the inclusion and exclusion criteria. After enrollment, participants were pseudonymized, and only pseudonymized data were used for further analyses. Of the 32 enrolled participants, 1 (3.1%) withdrew informed consent, and 3 (9.4%) additional participants were withdrawn by the investigator for medical or technical reasons (Figure 1). All 4 participants requested deletion of their study data in accordance with German law. Consequently, these data were removed from the study records and could not be included in the analyses. Another 3.1% (n=1) of the participants withdrew informed consent before the baseline assessment was performed.

### Recruitment Strategy

Patient databases at the University Hospital of Würzburg were screened for eligible patients with axSpA. These candidates were personally invited via phone by the investigators to participate in the study. At a screening appointment, eligibility criteria were verified by the primary investigator, and informed consent was obtained.

### Variables

The main outcomes of this substudy were the German versions of the uMARS and NPS. Both questionnaires were provided to participants in printed form by 2 medical students (AW and PP). Participants completed the questionnaires independently without any external influence. The German versions of both questionnaires used in the present study are provided in Multimedia Appendix 2. To explore potential determinants of app quality, user experience, and willingness to recommend the app, data on improvements in spinal mobility, assessed using the Bath Ankylosing Spondylitis Metrology Index (BASMI; metric scale; range 0-10), BASDAI (metric scale; range 0-10), and objectively tracked use behavior, were reused from the parent trial. Baseline BASDAI and demographic characteristics were included to examine whether app ratings differed according to patients’ initial disease status, treatment response, or characteristics. Use behavior included the absolute number of use days (days absolute), points collected on the app (points absolute), the proportion of use days relative to the maximum possible number of use days (percentage), and absolute use time in minutes. In addition to the baseline characteristics, no further data or analyses from the parent trial were double reported in the present study.

### Questionnaires

#### uMARS

The core of the uMARS consists of 20 items and represents a simplified version of the original 23-item Mobile App Rating Scale [13,22]. In this study, the translated German version of the questionnaire was used [13,23]. The uMARS is a validated instrument for assessing app quality and user experience across 5 dimensions or sections—engagement (A), functionality (B), aesthetics (C), information (D), and subjective quality (E)—each rated on a 5-point scale from 1 to 5, with 5 representing the maximum score [13,22]. The mean of the 4 domains’ mean scores (A-D) represents the uMARS overall app quality (objective quality), ranging from 1 to 5 [13,22]. For subjective quality, no compound score exists. The additional section F assesses perceived impact in 6 additional questions [13,22]. The complete questionnaire that was used in this study thereby consisted of a total of 26 questions.

#### NPS

Willingness to recommend the app was assessed using the NPS, which uses an 11-point numerical rating scale ranging from 0 to 10, with 10 representing the maximum [14,24]. Responses from 0 to 6 are classified as detractors, scores of 7 or 8 are classified as passives, and scores of 9 or 10 are classified as promoters [14]. The overall NPS is calculated by subtracting the proportion of detractors from the proportion of promoters [14,17]. The highest possible promoter rate is 100%, and the lowest is −100%. In addition, the mean numerical rating scale score of the NPS was reported.

### Data Sources

The pseudonymized paper-based questionnaires were collected by the 2 medical doctoral students (AW and PP) assisting the trial and were stored in the pseudonymized patient study files at the University Hospital of Würzburg. Paper questionnaires contained only the study identification number and no directly identifying information. After completion, they were stored as study source documents in the patients’ study records in accordance with institutional documentation requirements. All analyses were performed using pseudonymized study data. Values were extracted from the paper-based questionnaires and digitized for further evaluation.

### Study Size

Sample size calculation was performed for changes in the primary end point (BASMI) of the parent trial. A sample size of 24 was calculated for detecting significant improvements in the primary end point. A dropout rate of 20% was assumed, resulting in a total sample size of 30.

### Statistical Methods

#### General Considerations

Descriptive analyses were performed using means and SDs. Normality was assessed using the Shapiro-Wilk test. As the normality assumption was not met, correlations were analyzed using the Spearman rank correlation coefficient, and between-group differences were assessed using the Mann-Whitney *U* test. Medians and IQRs are reported for nonnormally distributed variables. A *P* value below .05 was considered statistically significant. No correction for multiple testing was applied because of the exploratory nature of the analyses.

#### Software Used for Statistical Analyses

Microsoft Excel (version 2408) was used for descriptive statistics and data storage. Correlation analyses, the Shapiro-Wilk test, and the Mann-Whitney *U* test were performed using Prism (version 5.0; GraphPad Software). Prism was also used for graphical visualizations of the results.

#### Missing Data

Given the exploratory nature of this substudy and the fact that the observed dropout rate was lower than the anticipated 20% used in the sample size calculation, no imputation of missing values was performed.

### Ethical Considerations

This study was conducted in accordance with the Declaration of Helsinki and good clinical practice guidelines. The study protocol was approved by the Ethics Committee of the Faculty of Medicine of the University of Würzburg (ID: DE/EKBY13; approval date: August 1, 2023; 93/23-am). Personal data of the participants were pseudonymized and processed according to institutional policies and applicable regulations. All participants gave written informed consent to take part in the study. Participants did not receive any financial compensation for taking part in the trial.

## Results

### Patient Flow and Characteristics

A total of 27 participants completed the survey. The study flow is shown in Figure 1. Patient characteristics—including the male-to-female ratio and the proportion of participants with radiographic axSpA—were evenly distributed. Detailed characteristics are shown in Table 1.

**Table 1:** Patient characteristics (N=27).

Characteristic				Values
Female sex, n (%)				13 (48.1)
Age (y), mean (SD; range)				49.1 (11.9; 22-66)
Radiographic axSpA^a^, n (%)				13 (48.1)
Nonradiographic axSpA, n (%)				14 (51.9)
BASDAI^b^ (0-10), mean (SD)				4.7 (1.7)
Biological or targeted synthetic DMARD^c^ therapy, n (%)				18 (66.7)
**Exercise and physical therapy engagement**				
	**Home-based exercise**			
		Participants, n (%)	12 (44.4)	
		Time spent (min per wk), mean (SD)	16.5 (19.9)	
	**Supervised group exercise therapy or sports**			
		Participants, n (%)	12 (44.4)	
		Time spent (min per wk), mean (SD)	25.7 (31.0)	
		Physiotherapy for ≤1 session per wk, n (%)	13 (48.1)	

^a^axSpA: axial spondyloarthritis.

^b^BASDAI: Bath Ankylosing Spondylitis Disease Activity Index.

^c^DMARD: disease-modifying antirheumatic drug.

### App Quality, User Experience, and Willingness to Recommend

The mean uMARS overall app quality score (objective quality) was 4.44 (SD 0.43). The highest-rated category was functionality (mean 4.54, SD 0.53), followed by aesthetics (mean 4.51, SD 0.41) and engagement (mean 4.39, SD 0.52). The lowest-rated category was information, with a mean score of 4.34 (SD 0.49). Figure 3 illustrates the distribution of all uMARS objective quality subcategory scores. The first 2 items (questions 1 and 2) in the entertainment section that assess the gamification factor of the app were given mean scores of 4.48 (SD 0.69) and 4.59 (SD 0.62), respectively. In section E (subjective quality), the highest-scoring item was the supposed use frequency with a mean score of 4.70 (SD 0.67), followed by recommendation with 4.52 (SD 0.69) and willingness to pay for the app with 3.52 (SD 1.31). The app was awarded 4.37 (SD 0.63) stars on average. Awareness and knowledge were the highest-rated items of section F (perceived impact), each with a mean score of 4.56 (SD 0.75 for awareness and SD 0.58 for knowledge). These were followed by intention to change (mean 4.48, SD 0.64), attitudes (mean 4.37, SD 0.74), behavior change (mean 4.33, SD 0.83), and help seeking (mean 4.22, SD 0.89). The mean NPS rating was 9.52 (SD 0.75), corresponding to an overall NPS of 93%. The complete scoring in the uMARS is presented in detail in Multimedia Appendix 3.

**Figure 3:**
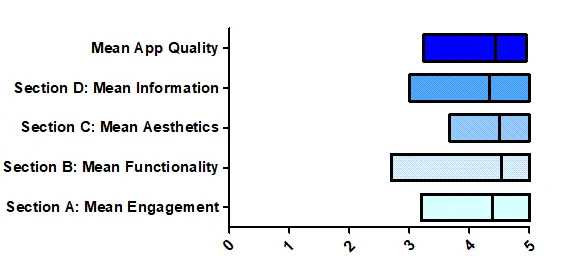
Total score on the user version of the Mobile App Rating Scale and scores on the separate domains. The boxes represent the range between the minimum and maximum values; the central line marks the mean.

### Correlation and Subgroup Analyses

The uMARS overall quality score showed a strong positive correlation with the NPS (ρ=0.57; *P*=.002). The NPS correlated negatively with the performance of physiotherapy (ρ=−0.64; *P*<.001) and sports at baseline (ρ=−0.42; *P*=.03). Only physiotherapy at baseline showed a similar negative correlation with the uMARS overall quality score (ρ=−0.66; *P*<.001). No correlations were observed between either the uMARS overall quality score or the NPS and age or the BASDAI at baseline (Multimedia Appendix 4). The median uMARS overall quality score did not differ between male (14/27, 51.9%) and female (13/27, 48.1%) participants (4.47, IQR 4.20-4.88 vs 4.55, IQR 4.10-4.75; *U*=63.50; *P*=.79), nor did the median NPS (9.5, IQR 9.0-10.0 vs 10, IQR 9.5-10.0; *U*=85.00; *P*=.12). The antirheumatic treatment at baseline also had no influence on the outcomes: the median uMARS overall quality score was similar in the group treated with biological or targeted synthetic disease-modifying antirheumatic drugs (18/27, 66.7%) compared to that without biological or targeted synthetic disease-modifying antirheumatic drug therapy (9/27, 33.3%; 4.52, IQR 4.23-4.88 vs 4.36, IQR 4.10-4.75; *U*=65.00; *P*=.43). No differences were observed for the NPS between both treatment groups (median 10, IQR 9.0-10.0 vs 10, IQR 9.0-10.0; *U*=79.00; *P*=.93).

Furthermore, NPS ratings and uMARS overall quality scores were not associated with changes in spinal mobility (BASMI) or disease activity (BASDAI). Similarly, objectively recorded app use, including the absolute number of use days, points collected within the app, the proportion of use days relative to the maximum possible number of use days, and total use time, showed no correlation with either the NPS or uMARS score (Multimedia Appendix 4).

## Discussion

### Summary of Main Findings

After 12 weeks of using Axia, participants reported high app quality and user experience on the uMARS, as well as high recommendation rates on the NPS. The NPS and uMARS quality mean score correlated negatively with the performance of physiotherapy and sports at baseline. Neither sex, medical treatment, nor age-related subgroup differences showed any meaningful association with uMARS quality mean score or NPS outcomes. The uMARS quality mean score and NPS ratings were independent of improvements in the primary and secondary outcomes or use behavior. This suggests that Axia is perceived as intuitive, appealing, and easy to use across all groups, supporting its suitability for a broad population of patients with axSpA.

### Detailed Discussion

The high uMARS and NPS scores suggest that disease-specific DTx may be acceptable tools for supporting home-based exercise in axSpA, for which regular exercise is strongly recommended but often difficult to maintain in routine care [2,4]. A digital intervention that is perceived as functional, engaging, and easy to use may help lower barriers to exercise by providing structured guidance outside supervised physiotherapy sessions [8]. Such tools may be particularly useful for patients with limited access to physiotherapy, patients living in rural areas, or patients who require flexible exercise options that can be integrated into daily life [4].

We previously evaluated user experience with the Axia app in an early usability test in 2023 using a self-designed but nonvalidated questionnaire over a 14-day test period [7]. The positive findings in this feasibility study could now be confirmed using the validated uMARS and NPS instruments.

The strong correlation between the uMARS overall score and the NPS, consistent with previous studies on DTx, supports the robustness of the observed findings [17]. The positive user experience observed in this study is likely explained by the combination of clinical effectiveness and user-centered design. Consistent with previous studies of Axia and systematic reviews and studies on DTx, intuitive functionality, engaging gamification, personalization, and demonstrated therapeutic benefit appear to be important drivers of sustained user engagement and DTx acceptance [7,9,12,25]. App ratings were independent of most patient characteristics at baseline, suggesting that the app was well designed for different patient subgroups within the axSpA spectrum. Interestingly, patients who were already physically active or regularly attended physiotherapy rated the app slightly less favorably, possibly reflecting a lower perceived need for additional exercise support. Importantly, user experience was not associated with the magnitude of clinical improvement, indicating that perceived app quality extends beyond treatment response alone. The comparatively low willingness-to-pay score may be explained by the fact that Axia was deliberately designed as a DHA, meaning that its costs are fully covered by statutory health insurance and patients do not have to make any out-of-pocket payments. Physiotherapy, which is an important comparator intervention to Axia, is also fully reimbursed by statutory health insurance in Germany for patients with axSpA.

An advantage of standardized questionnaires is that they enable comparisons across DTx. Compared with previously evaluated rheumatology DTx, including YogiTherapy and Rheuma-Auszeit, Axia achieved higher app quality and recommendation scores despite a substantially longer evaluation period [16,17,23]. In all these studies, app use was limited to a short period of minutes [16,17,23]. The participants in our study, however, used Axia for 12 weeks. Longer use periods typically increase the likelihood of technical issues, attrition, or detection of software bugs. Nevertheless, user experience remained consistently positive throughout the study, suggesting that high user satisfaction can be sustained beyond the initial novelty phase and supporting the feasibility of long-term implementation in routine care.

ViViRA is an official DHA according to German law that is approved for nonspecific low back pain [11]. As there has not yet been an approved and reimbursable DHA for inflammatory back pain in axSpA, ViViRA, developed for mechanical back pain, has often been prescribed [9,26-29]. HelloBetter Chronische Schmerzen is a DHA approved in Germany for patients with chronic pain conditions [18]. Both DHAs have reported lower NPS and app quality ratings than those observed for Axia [18,28,29]. Direct comparisons should, however, be interpreted cautiously because of differences in study populations and interventions.

The results also have implications for the implementation of reimbursed DTx in routine rheumatology care as DHAs are prescribable and reimbursed DTx in Germany and recent data indicate substantial interest in DHAs among patients with rheumatic diseases despite limited real-world adoption [30]. Patient acceptance is a prerequisite for successful integration into routine care [12]. However, successful implementation in routine care requires additional factors beyond regulatory approval and reimbursement, including clinical acceptance, organizational change, stakeholder communication, and integration into existing care pathways [31]. The comparatively low willingness-to-pay score further underlines the importance of reimbursement structures for equitable access to digital interventions, particularly because equitable implementation of digital health in rheumatology requires attention to access, infrastructure, digital literacy, and socioeconomic barriers [32].

### Limitations

This study’s single-center design and small sample size limit the generalizability of its findings. Because most participants were recruited from the same institution where the app was developed and previously tested, existing patient-physician relationships may have influenced responses and introduced selection bias, although similar bias exists in comparable studies. Furthermore, eHealth literacy was not assessed in this study, and therefore, potential effects of prior digital experience or technological proficiency on app perception and satisfaction could not be evaluated (potential selection bias). The 12-week evaluation period is longer than in most uMARS or NPS studies. While this allowed for comprehensive testing, it may limit comparability. Conversely, the extended duration can also be considered a strength as long-term use is more likely to reveal technical issues or declining engagement—yet the app maintained high ratings. Finally, the study included only German-speaking participants with a predefined digital literacy level, and the app is available only in German, which restricts external validity and may represent a further selection bias. Future multicenter trials with more diverse cohorts are needed to improve generalizability.

### Conclusions

In conclusion, Axia achieved high app quality and user experience ratings, as well as a high willingness to recommend the app after 12 weeks of use. The findings support growing evidence that disease-specific DTx with intuitive design and gamification can achieve high patient acceptance in axSpA. More broadly, they suggest that user-centered design, technical robustness, and behavioral engagement strategies are key factors for the successful implementation of DTx in routine rheumatology care.

## Data Availability

The source data are not kept in publicly available storage but can be available on reasonable request from the corresponding author.

## Appendix

Multimedia Appendix 1 STROBE checklist.

Multimedia Appendix 2 Questionnaires.

Multimedia Appendix 3 Table showing results for the user version of the Mobile App Rating Scale.

Multimedia Appendix 4 Correlation analyses.

## Abbreviations

ASAS: Assessment of SpondyloArthritis International Society
axSpA: axial spondyloarthritis
BASDAI: Bath Ankylosing Spondylitis Disease Activity Index
BASMI: Bath Ankylosing Spondylitis Metrology Index
DHA: digital health application
DTx: digital therapeutic
NPS: net promoter score
RCT: randomized controlled trial
STROBE: Strengthening the Reporting of Observational Studies in Epidemiology
uMARS: user version of the Mobile App Rating Scale
