# Supplementary material for: Assessment of App Quality, User Experience, and Willingness to Recommend a Digital Therapeutic for Patients With Axial Spondyloarthritis: Subanalysis of a 12-Week Pretest-Posttest Intervention Study
Source: JMIR Form Res. 2026 Oct 26;10:e88949. doi: 10.2196/88949 (PMC13749026; doi:10.2196/88949)
Supplement: Supplementary file 2 [file formative_v10i1e88949_app2.docx]

**Multimedia Appendix 2 to “High user satisfaction and recommendation rates of a digital exercise and disease management app for patients with axial spondyloarthritis: Subanalysis of a 12-week pre-post intervention study”**

**Authors: Hannah Labinsky ^1^, Amelie Wüst ², Patricia Possler ^2^, Tobias Heusinger ^2^, Maxime le Maire ^2^, Anna Fleischer ^3^, Karsten Sebastian Luetkens ^4^, Michael Gernert ^1^, Ottar Gadeholt ^1^, Robert Leppich ^5^, Astrid Schmieder ^6^, Ludwig Hammel ^7^, Billy Sperlich ^8^, Matthias Froehlich ^1^, Marc Schmalzing ^1^, and Patrick-Pascal Strunz ^1^.**

**Affiliations**

^1^ University Hospital Würzburg, Department of Internal Medicine 2, Rheumatology/Clinical Immunology, Würzburg, Germany

^2^ University of Würzburg, Medical Faculty, Würzburg, Germany

^3^ University Hospital Würzburg, Department of Internal Medicine 2, Psychosomatic Medicine, Würzburg, Germany

^4^ University Hospital Würzburg, Department of Diagnostic and Interventional Radiology, Würzburg, Germany

^5^ Chair of Software Engineering (Informatik II), Department of Computer Science, University of Würzburg, Würzburg, Germany

^6^ University Hospital Würzburg, Department of Dermatology, Venereology, and Allergology, Würzburg, Germany

^7^ Deutsche Vereinigung Morbus Bechterew e. V., Schweinfurt, Germany.

^8^ Integrative and Experimental Exercise Science and Training, Institute for Sports Science, University of Wuerzburg. Würzburg, Germany.

**Corresponding authors:**

**Patrick-Pascal Strunz, MD**

**University Hospital Würzburg**

**Department of Internal Medicine 2, Rheumatology/Clinical Immunology**

**Email:** [**Strunz_p@ukw.de**](mailto:Strunz_p@ukw.de)

**Used Questionnaires in the validated German version:**

**NET Promoter Score**

*Wie wahrscheinlich ist es, dass Sie die Axia-App einer Freundin/ einem Freund oder einer Kollegin/ einem Kollegen weiterempfehlen? Bitte kreuzen Sie das entsprechende Kästchen mit der Zahl an.*

**uMARS** (= User version Mobile Application Rating Scale)

**App Qualitätsbewertung**

Das MARS Rating erfasst die Qualität von Apps auf 4 Dimensionen. Alle Fragen werden von 1 „Mangelhaft“ bis 5 „Exzellent“ bewertet. Bitte markieren Sie die Nummer die Sie am zutreffendsten empfinden. Bitte benutzen Sie die Beschreibung welche in jeder Kategorie zur Verfügung gestellt werden.

**SECTION A**

**Engagement – Spaß, Interesse, individuell Anpassbarkeit, Interaktivität (z.B. sendet Alarme, Nachrichten, Erinnerungen, Feedback, Austausch mit anderen ist möglich), Zielgruppenspezifität**

I. **Unterhaltung: ist die App unterhaltsam und lustig zu benutzen? Werden Strategien zur Steigerung des Engagements durch Unterhaltung (z.B. Gamification) geboten?**
1. Langweilig, gar nicht unterhaltsam
2. Überwiegend langweilig
3. OK, unterhaltsam genug um sich für eine kurz Zeit mit der App zu beschäftigen (<5 Minuten)
4. Mittelmäßig lustig und unterhaltsam, würde einen User für einige Zeit unterhalten (5 – 10 Minuten)
5. Sehr unterhaltsam und lustig, regt zu mehrmaligen Benutzen an
II. **Interesse: Ist die App interessant zu benutzen? Werden Strategien angewandt um das Engagement durch eine interessante Präsentation der Inhalte zu erhöhen?**
1. Überhaupt nicht interessant
2. Überwiegend nicht interessant
3. OK, weder interessant noch uninteressant; Nutzer würden sich damit eine kurze Zeit beschäftigen (<5 Minuten)
4. Mittelmäßig interessant; Nutzer würden sich damit einige Zeit beschäftigen (insgesamt 5 – 10 Minuten)
5. Überaus interessant, so dass man zur wiederholten Nutzung der App angeregt wird
III. **Individuelle Anpassbarkeit: Können alle nötigen Einstellungen den eigenen Wünschen entsprechend angepasst werden und werden diese Anpassungen abgespeichert (z.B. Klänge, Inhalt und Benachrichtigungen)?**
1. Individuelle Anpassungen sind nicht möglich oder es ist nötig, diese bei jeder Nutzung neu den eigenen Wünschen anzupassen
2. Einige wenige individuelle Anpassungen sind möglich, aber durch die nur wenigen Möglichkeiten ist die Funktionalität der App beeinträchtigt
3. Grundlegende Einstellungen können individuell angepasst werden, so dass die Funktionalität der App gewährleistet ist
4. Vielfältige Optionen zur individuellen Anpassbarkeit stehen zur Verfügung
5. Eine maßgeschneiderte Anpassung an die Charakteristika und Wünsche der Nutzer ist möglich und alle individuellen Anpassungen werden für zukünftige Nutzungen übernommen
IV. **Interaktivität: Werden Nutzereingaben, Feedback und Aufforderungen (Erinnerungen, Optionen zum Austausch mit anderen, Benachrichtigungen, etc.) gegeben? Bemerkung: Diese Funktionen müssen individuell anpassbar und nicht überfördernd sein.**
1. Keine interaktiven Funktionen und/oder keine Eingaben der Nutzer sind möglich
2. Unzureichende Interaktivität, oder Feedback, oder Optionen für Eingaben der User, limitierte Funktionalität
3. Grundlegende interaktive Funktionen, angemessene Funktionalität
4. Vielfältige interaktive Funktionen, Feedback und Optionen für Eingaben der Nutzer sind vorhanden
5. Interaktive Funktionen, Feedback und Optionen für Eingaben der Nutzer sind auf einem so hohen Niveau, dass man das Gefühl hat, die App stellt sich auf die Person des Nutzers ein
V. **Zielgruppe: Ist der Inhalt der App (visuelle Aufbereitung, Sprache, Design) passend um die Zielgruppe anzusprechen?**
1. Sehr unpassend/unklar/verwirrend
2. Eher unpassend/unklar/verwirrend
3. OK, aber nicht Zielgruppen orientiert. Kann unpassend/unklar/verwirrend sein
4. Zielgruppenspezifisch, mit kleinen Mängeln
5. Sehr zielgruppenspezifisch, keine Probleme

**A: Engagement Mittelwert = ______________________**

**SECTION B**

**Funktionalität – Funktionen der App, Usability, Navigation, logischer Aufbau und motorische, gestische Handhabung der App**

VI. **Leistung: Wie akkurat, schnell funktionieren die Komponenten (Knöpfe, Menüs) und Funktionen der App?**
1. Die App funktioniert nicht. Keine oder unzureichende Antwort (z.B. Abbrüche, Fehler, nicht funktionierende Funktionen, etc.)
2. Einige Funktionen laufen, jedoch langsam oder größere technische Probleme
3. App funktioniert im Allgemeinen. Einige technische Probleme oder gelegentlich langsam
4. Die App funktioniert insgesamt gut, mit kleineren, vernachlässigbaren Problemen
5. Die App arbeitet fehlerfrei und schnell; keine technischen Fehler, oder – falls zutreffend – enthält einen Fortschrittsbalken
VII. **Usability: Wie leicht ist es den Umgang mit der App zu erlernen? Wie klar sind die Benennungen der Menüs, Symbole und Instruktionen?**
1. Keine oder unzureichende Erklärungen; Menübeschriftungen und Symbole sind verwirrend und/oder kompliziert
2. Nach hohem Aufwand benutzbar
3. Nach einigem Aufwand benutzbar
4. Benutzung ist leicht erlernbar oder die App weist klare Instruktionen zur Benutzung auf
5. Eine intuitive und einfache Benutzung der App ist sofort möglich
VIII. **Navigation: Ist die Menüführung logisch, akkurat, passend und wird nicht unterbrochen; sind alle notwenigen Verbindungen zwischen den Sektionen vorhanden?**
1. Verschiedene Abschnitte wirken unzusammenhängend/zufällig/verwirrend. Die Navigation ist kompliziert
2. Nach geraumer Zeit und hohem Aufwand benutzbar
3. Nach einiger Zeit und einigem Bemühen benutzbar
4. Leicht zu bedienen; es fehlen vernachlässigbare Verknüpfungen
5. Sehr logisch, einfach, klar und intuitive Menüführung. Bietet Verknüpfungen zum schnellen Wechsel zwischen verschiedenen Bereichen/Menüpunkten an
IX. **Motorisches, gestisches Design: Sind Interaktionen (tippen, wischen, drücken, scrollen, zoomen) einheitlich und intuitiv über alle Komponenten und Ansichten?**
1. Überhaupt nicht einheitlich; verwirrend
2. Häufig nicht einheitlich; verwirrend
3. OK mit einigen uneinheitlichen/verwirrenden Elementen
4. Häufig einheitlich/intuitiv mit vernachlässigbaren Problemen
5. Auf perfekte Weise einheitlich und intuitiv verständlich

**B: Funktionalität Mittelwert = ______________________**

**SECTION C**

**Ästhetik – Graphisches Design, visueller Anreiz, farbliche Gestaltung und stilistische Einheit**

X. **Layout: Sind das Arrangement und die Größe der Knöpfe/ Symbole/ Menüführung/ Inhalt passend oder entsprechend zoombar?**
1. Mangelhaftes Design – unordentlich/ungeordnet; einige Optionen können nicht ausgewählt, gefunden, gesehen oder gelesen werden; Bildschirm wird nicht optimal genutzt
2. Schwaches Design – nicht einheitlich, unklar; einige Optionen sind schwierig auszuwählen, zu finden, zu sehen und zu leben
3. Mittelmäßige Qualität des Designs – einige Probleme Optionen auszuwählen, zu finden, zu sehen und zu lesen
4. Gutes Design – überwiegend klar; Optionen können ausgewählt, gefunden, gesehen und gelesen werden
5. Professionelles Design – einfach, klar, übersichtlich, logisch organisiert; Bildschirm wird optimal genutzt; jede einzelne Komponente des Designs erfüllt seinen Zweck
XI. **Grafik: Wie hoch ist die Qualität und die Auflösung der Grafiken von Knöpfen, Symbolen, Menüführung und Inhalten?**
1. Grafiken wirken amateurhaft, schlechtes visuelles Design – völlig unproportional, stilistisch nicht einheitlich
2. Niedrige Qualität (z.B. Auflösung) der Grafiken. Niedrige Qualität des visuellen Designs – unproportional und stilistisch nicht einheitlich
3. Mittelmäßige Qualität der Grafiken und des visuellen Designs – überwiegend einheitlich im Design
4. Hohe Qualität der Grafiken und gute Auflösung des visuellen Designs – überwiegend proportional und einheitlich im Stil
5. Sehr hohe Qualität der Grafiken und sehr gute Auflösung des visuellen Designs – durchgehend proportional und einheitlich im Stil
XII. **Visueller Anreiz: Wie gut sieht die App aus?**
1. Kein visueller Anreiz – unangenehm zu betrachten, schwaches Design, beißende oder nicht zusammen passende Farben
2. Wenig visueller Anreiz – schlechtes Design, schlechter Farbgebrauch, visuell langweilig
3. Etwas visueller Anreiz – durchschnittlich weder angenehm noch unangenehm
4. Hoher visueller Anreiz – makellose Grafiken, einheitliches und professionelles Design
5. Wie in Punkt 4 beschrieben, darüber hinaus: Überaus großer visueller Anreiz – einprägsam, sticht positiv heraus; Farbgestaltung wirkt sich positiv auf die Optionen und die Menüführung der App aus

**C: Ästhetik Mittelwert = ______________________**

**SECTION D**

**Information – Beinhaltet Informationen mit hoher Güte, Qualität (z.B. Texte, Feedback, Messinstrumente, Messergebnisse, Hinweise auf Literatur zum Vertiefen) aus einer glaubwürdigen Quelle. Bitte wählen die N/A (nicht anwendbar) aus, wenn der Punkt unzutreffend ist.**

XIII. **Qualität der Information: Ist der Inhalt korrekt, gut verfasst, relevant für das Ziel und das Thema der App?**

N/A: Die App enthält keine Informationen

1. Irrelevant, ungeeignet, unzusammenhängend, nicht korrekt
2. Kaum relevant, kaum geeignet, kaum zusammenhängend, möglicherweise nicht korrekt
3. Mittelmäßig relevant, mittelmäßig geeignet, mittelmäßig zusammenhängend, wirkt korrekt
4. Relevant, geeignet, zusammenhängend, korrekt
5. Sehr Relevant, sehr geeignet, sehr zusammenhängend, sehr korrekt
XIV. **Quantität der Informationen: Ist der Inhalt der App umfassend und dennoch prägnant?**

N/A: App beinhaltet keine Information

1. Minimal oder überfordernd
2. Unzureichend oder möglicherweise überfordernd
3. OK, aber weder umfangreich noch prägnant
4. Bietet eine große Menge an Informationen, hat Lücken oder unnötige Details oder bietet keine weiteren Hinweise auf Literatur zum Vertiefen
5. Umfangreich und prägnant; beinhaltet Hinweise auf Literatur zum Vertiefen
XV. **Visuelle Informationen: Sind die visuellen Erklärungen von Konzepten – anhand von Tabellen, Graphiken, Bilder, Videos etc. – klar, logisch, korrekt?**

N/A: App beinhaltet keine visuellen Information (beinhaltet z.B. nur Audio-Dateien oder Text)

1. Vollkimmen unklar/ verwirrend/ falsch oder visuelle Informationen sind eigentlich nötig, fehlen aber
2. Überwiegend unklar/ verwirrend/ falsch
3. OK, aber oft unklar/ verwirrend/ falsch
4. Meistens klar, logisch und richtig, mit unerheblichen/ vernachlässigbaren Problemen
5. Perfekt klar, logisch und richtig
XVI. **Glaubwürdigkeit: Kommt die App aus einer legitimen Quelle (ist dies aus der Appstore-Beschreibung oder in der App selbst zu entnehmen)?**
1. Die Quelle ist bekannt aber die Legitimität/ Glaubwürdigkeit der Quelle ist fragwürdig (z.B. Werbe/ Handelsbranche mit eigennützigem Interesse)
2. Scheint von einer legitimen Quelle zu kommen, kann aber nicht nachgeprüft werden (z.B. keine Webseite)
3. Entwickelt von einer kleinen gemeinnützigen Organisation (NGO: z.B. Krankenhaus, Zentrum etc.) oder von einer spezialisierten wirtschaftlichen Firma oder von Fördermittelgeber
4. Von der Regierung, einer Universität oder einer ähnlichen Institution entwickelt
5. Entwickelt mit Hilfe von wettbewerbsorientierten Regierungs- oder Forschungsgeldern (z.B. DFG, BMBF)

**D: Information Mittelwert = ______________________ ***

* Fragen die mit „Nicht verfügbar/ keine Antwort“ geratet wurden, nicht in die Mittelwert-Berechnung miteinbeziehen.

**Subjektive App Qualität**

**SECTION E**

XVII. **Würden Sie die App Personen empfehlen die davon profitieren könnten?**
1. Überhaupt nicht:	Ich würde die App niemanden empfehlen
2. Ich würde die App nur wenigen Personen empfehlen
3. Vielleicht:	Ich würde die App schon einigen Personen empfehlen
4. Ich würde die App vielen Personen empfehlen
5. Auf jeden Fall:	Ich würde die App jedem empfehlen
XVIII. **Wie viele Male würden Sie die App in den nächsten zwölf Monaten nutzen, falls diese relevant für Sie wäre?**
1. Nie
2. 1 – 2
3. 3 – 10
4. 10 – 50
5. > 50
XIX. **Würden Sie diese App erwerben, wenn Sie kostenpflichtig wäre?**
1. Nein
2. Vielleicht
3. Ja
XX. **Wie viele Sterne würden Sie für die App insgesamt vergeben**
1. ★	Eine der schlechtesten Apps, die ich je genutzt habe
2. ★★
3. ★★★	Durchschnittlich
4. ★★★★
5. ★★★★★	Eine der besten Apps, die ich je genutzt habe

**Wahrgenommene Auswirkungen**

**SECTION F**

**Diese zusätzlichen Angaben erfassen die wahrgenommen Auswirkungen der App-Nutzung auf das Wissen, die Einstellung und Veränderungsintention sowie die Wahrscheinlichkeit einer tatsächlichen Veränderung des Verhaltens hin zum gewünschten Gesundheitsverhalten.**

1. **Bewusstsein: Die App steigert das Bewusstsein für die Wichtigkeit, sich mit dem Gesundheitsverhalten zu beschäftigen**

1	2	3	4	5

Stimme überhaupt nicht zu	Stimme sehr zu

2. **Wissen: Die App erhöht das Verständnis und Wissen über das gewünschte Gesundheitsverhalten**

1	2	3	4	5

Stimme überhaupt nicht zu	Stimme sehr zu

3. **Einstellung: Die App ändert die Einstellung, das Gesundheitsverhalten zu verbessern**

1	2	3	4	5

Stimme überhaupt nicht zu	Stimme sehr zu

4. **Absicht zur Verhaltensänderung: Die App fördert die Absicht und Motivation, das Gesundheitsverhalten zu verbessern**

1	2	3	4	5

Stimme überhaupt nicht zu	Stimme sehr zu

5. **Suche nach Hilfe: Die App ermutigt dazu, sich weiter Hilfen bezüglich des Gesundheitsverhaltens (wenn erforderlich) zu suchen**

1	2	3	4	5

Stimme überhaupt nicht zu	Stimme sehr zu

6. **Verhaltensänderung: Die App ermöglicht die gewünschte Änderung des Gesundheitsverhaltens**

1	2	3	4	5

Stimme überhaupt nicht zu	Stimme sehr zu
