# Supplementary material for: Assessment of App Quality, User Experience, and Willingness to Recommend a Digital Therapeutic for Patients With Axial Spondyloarthritis: Subanalysis of a 12-Week Pretest-Posttest Intervention Study
Source: JMIR Form Res. 2026 Oct 26;10:e88949. doi: 10.2196/88949 (PMC13749026; doi:10.2196/88949)
Supplement: Supplementary file 3 [file formative_v10i1e88949_app3.docx]

**Multimedia Appendix 3 to “High user satisfaction and recommendation rates of a digital exercise and disease management app for patients with axial spondyloarthritis: Subanalysis of a 12-week pre-post intervention study”**

**Authors: Hannah Labinsky ^1^, Amelie Wüst ², Patricia Possler ^2^, Tobias Heusinger ^2^, Maxime le Maire ^2^, Anna Fleischer ^3^, Karsten Sebastian Luetkens ^4^, Michael Gernert ^1^, Ottar Gadeholt ^1^, Robert Leppich ^5^, Astrid Schmieder ^6^, Ludwig Hammel ^7^, Billy Sperlich ^8^, Matthias Froehlich ^1^, Marc Schmalzing ^1^, and Patrick-Pascal Strunz ^1^.**

**Affiliations**

^1^ University Hospital Würzburg, Department of Internal Medicine 2, Rheumatology/Clinical Immunology, Würzburg, Germany

^2^ University of Würzburg, Medical Faculty, Würzburg, Germany

^3^ University Hospital Würzburg, Department of Internal Medicine 2, Psychosomatic Medicine, Würzburg, Germany

^4^ University Hospital Würzburg, Department of Diagnostic and Interventional Radiology, Würzburg, Germany

^5^ Chair of Software Engineering (Informatik II), Department of Computer Science, University of Würzburg, Würzburg, Germany

^6^ University Hospital Würzburg, Department of Dermatology, Venereology, and Allergology, Würzburg, Germany

^7^ Deutsche Vereinigung Morbus Bechterew e. V., Schweinfurt, Germany.

^8^ Integrative and Experimental Exercise Science and Training, Institute for Sports Science, University of Wuerzburg. Würzburg, Germany.

**Corresponding authors:**

**Patrick-Pascal Strunz, MD**

**University Hospital Würzburg**

**Department of Internal Medicine 2, Rheumatology/Clinical Immunology**

**Email:** [**Strunz_p@ukw.de**](mailto:Strunz_p@ukw.de)

| **Scales and items** | **Frequencies the scored points** | | | | | **Mean score (SD)** |
| --- | --- | --- | --- | --- | --- | --- |
|  | **1** | **2** | **3** | **4** | **5** |  |
| **A: Engagement** | | | | | | |
| I. Entertainment | 0 | 0 | 3 | 8 | 16 | 4.48 (0.69) |
| II. Interest | 0 | 0 | 2 | 7 | 18 | 4.59 (0.62) |
| III. Customization | 0 | 2 | 3 | 14 | 8 | 4.04 (0.84) |
| IV. Interactivity | 0 | 1 | 7 | 9 | 10 | 4.04 (0.88) |
| V. Target group | 0 | 0 | 0 | 6 | 21 | 4.78 (0.42) |
| Mean Engagement | | | | | | 4.39 (0.52) |
| **B: Functionality** | | | | | | |
| VI. Performance | 0 | 1 | 1 | 12 | 13 | 4.37 (0.73) |
| VII. Ease of use | 0 | 0 | 3 | 8 | 16 | 4.48 (0.69) |
| VIII. Navigation | 0 | 0 | 2 | 5 | 20 | 4.67 (0.61) |
| IX. Gestural design / flow logic | 0 | 0 | 1 | 10 | 16 | 4.56 (0.57) |
| Mean Functionality | | | | | | 4.54 (0.53) |
| **C: Aesthetics** | | | | | | |
| X. Layout | 0 | 0 | 0 | 14 | 13 | 4.48 (0.50) |
| XI. Graphics / Visual appeal | 0 | 0 | 0 | 8 | 19 | 4.70 (0.46) |
| XII. Overall aesthetic quality | 0 | 0 | 1 | 14 | 12 | 4.41 (0.63) |
| Mean Aesthetics | | | | | | 4.51 (0.41) |
| **D: Information** | | | | | | |
| XIII. Quality of information | 0 | 0 | 2 | 11 | 14 | 4.44 (0.63) |
| XIV. Quantity of information | 0 | 0 | 1 | 12 | 14 | 4.48 (0.57) |
| XV. Visual information | 0 | 0 | 1 | 12 | 14 | 4.48 (0.57) |
| XVI. Credibility of source | 0 | 1 | 6 | 13 | 7 | 3.96 (0.79) |
| Mean Information | | | | | | 4.34 (0.49) |
| **Quality Mean Score** | | | | | | **4.44 (0.43)** |
| **E: Subjective Quality** | | | | | | |
| XVII. Would you recommend this app to people who might benefit from it | 0 | 0 | 4 | 5 | 18 | 4.52 (0.69) |
| XVIII. How many times do you think you would use this app in the next 12 months if you needed it? | 0 | 1 | 0 | 5 | 21 | 4.70 (0.67) |
| XIX. Would you pay for this app? | 3 | 0 | 14 | 0 | 10 | 3.52 (1.31) |
| XX. What is your overall rating of the app? | 0 | 0 | 2 | 13 | 12 | 4.37 (0.63) |
| **F: Perceived Impact** | | | | | | |
| F1. Awareness – This app has increased my awareness of the importance of addressing the health  behaviour | 0 | 0 | 4 | 4 | 19 | 4.56 (0.75) |
| F2. Knowledge – This app has increased my knowledge/understanding of the health behaviour | 0 | 0 | 1 | 10 | 16 | 4.56 (0.58) |
| F3. Attitudes – The app has changed my attitudes toward improving this health behaviour | 0 | 1 | 1 | 12 | 13 | 4.37 (0.74) |
| F4. Intention to change – The app has increased my intentions/motivation to address this health  behaviour | 0 | 0 | 2 | 10 | 15 | 4.48 (0.64) |
| F5. Help seeking – This app would encourage me to seek further help to address the health  behaviour (if I needed it) | 0 | 1 | 4 | 10 | 12 | 4.22 (0.89) |
| F6. Behaviour change – Use of this app will increase/decrease the health behaviour | 0 | 0 | 6 | 6 | 15 | 4.33 (0.83) |

**Supplementary Table 1: Distribution of the frequencies of the scored points in each uMARS subscale in detail**

The five uMARS categories and the subscale F with their corresponding items are displayed on the left. On the right, the frequency of scores for each item as well as the mean and standard deviation (SD) are presented. The Quality Mean Score is calculated as the mean of each individual Quality Mean Score, rather than as the mean of the category means shown.
