# Supplementary material for: Assessment of App Quality, User Experience, and Willingness to Recommend a Digital Therapeutic for Patients With Axial Spondyloarthritis: Subanalysis of a 12-Week Pretest-Posttest Intervention Study
Source: JMIR Form Res. 2026 Oct 26;10:e88949. doi: 10.2196/88949 (PMC13749026; doi:10.2196/88949)
Supplement: Supplementary file 4 [file formative_v10i1e88949_app4.docx]

**Multimedia Appendix 4 to “High user satisfaction and recommendation rates of a digital exercise and disease management app for patients with axial spondyloarthritis: Subanalysis of a 12-week pre-post intervention study”**

**Authors: Hannah Labinsky ^1^, Amelie Wüst ², Patricia Possler ^2^, Tobias Heusinger ^2^, Maxime le Maire ^2^, Anna Fleischer ^3^, Karsten Sebastian Luetkens ^4^, Michael Gernert ^1^, Ottar Gadeholt ^1^, Robert Leppich ^5^, Astrid Schmieder ^6^, Ludwig Hammel ^7^, Billy Sperlich ^8^, Matthias Froehlich ^1^, Marc Schmalzing ^1^, and Patrick-Pascal Strunz ^1^.**

**Affiliations**

^1^ University Hospital Würzburg, Department of Internal Medicine 2, Rheumatology/Clinical Immunology, Würzburg, Germany

^2^ University of Würzburg, Medical Faculty, Würzburg, Germany

^3^ University Hospital Würzburg, Department of Internal Medicine 2, Psychosomatic Medicine, Würzburg, Germany

^4^ University Hospital Würzburg, Department of Diagnostic and Interventional Radiology, Würzburg, Germany

^5^ Chair of Software Engineering (Informatik II), Department of Computer Science, University of Würzburg, Würzburg, Germany

^6^ University Hospital Würzburg, Department of Dermatology, Venereology, and Allergology, Würzburg, Germany

^7^ Deutsche Vereinigung Morbus Bechterew e. V., Schweinfurt, Germany.

^8^ Integrative and Experimental Exercise Science and Training, Institute for Sports Science, University of Wuerzburg. Würzburg, Germany.

**Corresponding authors:**

**Patrick-Pascal Strunz, MD**

**University Hospital Würzburg**

**Department of Internal Medicine 2, Rheumatology/Clinical Immunology**

**Email:** [**Strunz_p@ukw.de**](mailto:Strunz_p@ukw.de)

| **Characteristic** | **Net Promoter Score** | **uMARS Quality mean score** |
| --- | --- | --- |
| **Delta BASMI** | 0.14 | -0.26 |
| **Delta BASDAI** | -0.16 | -0.27 |
| **HbE at BL** | 0.11 | 0.11 |
| **Functional Training/ Sports at BL** | -0.42* | -0.14 |
| **Physiotherapy at BL** | -0.64*** | -0.66*** |
| **Age at BL** | -0.11 | 0.12 |
| **BASDAI at BL** | -0.04 | -0.04 |
| **Days used total** | 0.07 | 0.00 |
| **Minutes absolute** | 0.16 | 0.26 |
| **Points collected in the app** | 0.17 | 0.18 |
| **Percentage of used days on all usage days** | 0.03 | 0.00 |

**Supplementary Table 2: Correlation matrix**

BASDAI, Bath ankylosing spondylitis disease activity index; BASMI, Bath ankylosing spondylitis metrology index; BL, baseline; HbE, home-based exercise. *: p<0.05, ***: p<0.001
